# Sleep Apnea Syndrome: Prevalence and Comorbidity with Other Non-communicable Diseases and HIV Infection, among Hospitalized Patients in Yaoundé, Cameroon

**DOI:** 10.1155/2022/4359294

**Published:** 2022-02-10

**Authors:** Massongo Massongo, Leonard Ngarka, Dodo Adamou Balkissou, Virginie Poka-Mayap, Steve Voufouo Sonwa, Godwin Y. Tatah, Leonard N. Nfor, Michel K. Mengnjo, Eric-Samuel Chokoke, Ben Patrick Michel Moutlen, Stephen Perrig, Eric Walter Pefura-Yone, Alfred Kongnyu Njamnshi

**Affiliations:** ^1^Department of Internal Medicine and Specialties, Faculty of Medicine and Biomedical Sciences, The University of Yaoundé I, Yaoundé, Cameroon; ^2^Pulmonology Department, Yaoundé Jamot Hospital, Yaoundé, Cameroon; ^3^Brain Research Africa Initiative (BRAIN), Yaoundé, Cameroon; ^4^Brain Research Africa Initiative (BRAIN), Geneva, Switzerland; ^5^Faculty of Medicine of Garoua, The University of Ngaoundere, Garoua, Cameroon; ^6^Faculty of Medicine and Biomedical Sciences, The University of Yaoundé I, Yaoundé, Cameroon; ^7^Neurology Department, Yaoundé Central Hospital, Yaoundé, Cameroon; ^8^Sleep Studies Laboratory, Geneva University Hospitals, Geneva, Switzerland

## Abstract

**Background:**

Sleep apnea syndrome (SAS), a growing public health threat, is an emerging condition in sub-Saharan Africa (SSA). Related SSA studies have so far used an incomplete definition. This study is aimed at assessing SAS using an American Academy of Sleep Medicine (AASM) complete definition and at exploring its relationship with comorbidities, among patients hospitalized in a Cameroonian tertiary hospital.

**Methods:**

This cross-sectional study was conducted in cardiology, endocrinology, and neurology departments of the Yaoundé Central Hospital. Patients aged 21 and above were consecutively invited, and some of them were randomly selected to undergo a full night record using a portable sleep monitoring device, to diagnose sleep-disordered breathing (SDB). SAS was defined as an apnea − hypopnea index (AHI) ≥ 5/h, associated with either excessive daytime sleepiness or at least 3 compatible symptoms. Moderate to severe SAS (MS-SAS) stood for an AHI ≥ 15/h. We used chi-square or Fisher tests to compare SAS and non-SAS groups. *Findings*. One hundred and eleven patients presented a valid sleep monitoring report. Their mean age ± standard deviation (range) was 58 ± 12.5 (28–87) years, and 53.2% were female. The prevalence (95% confident interval (CI)) of SAS was 55.0 (45.7, 64.2)% and the one of MS-SAS 34.2 (25.4, 43.1)%. The obstructive pattern (90.2% of SAS and 86.8% of MS-SAS) was predominant. The prevalence of SAS among specific comorbidities ranged from 52.2% to 75.0%. Compared to SAS free patients, more SAS patients presented with hypertension (75.4% vs. 48.0%, *p* = 0.005%), history of stroke (36.7% vs. 32.0%, *p* = 0.756), cardiac failure (23.0% vs. 12.0%, *p* = 0.213), and combined cardiovascular comorbidity (80.3% vs. 52.0%, *p* = 0.003). Similar results were observed for MS-SAS. Metabolic and neuropsychiatric comorbidities did not differ between SAS and SAS-free patients.

**Conclusion:**

The SAS diagnosed using modified AASM definition showed high prevalence among patients hospitalized for acute medical conditions, as it was found with SDB. Unlike HIV infection, metabolic and brain conditions, cardiovascular comorbidities (hypertension and cardiac failure) were significantly more prevalent in SAS patients.

## 1. Introduction

Sleep apnea syndrome (SAS) is a growing public health threat worldwide, not only in the high-income countries (HIC) where its consequences have been largely studied [1–10]. In sub-Saharan Africa (SSA), SAS appears as an emerging disease, with few epidemiological studies published so far. Most of these studies come from Nigeria and Cameroon, and address the high risk of obstructive SAS (HR-OSAS) based on validated questionnaires (STOPBANG and Berlin especially). The HR-OSAS rate ranges 17.4%–36.6% in the general adult population [11–14] and reaches 48.8% among commercial drivers in Nigeria [15]. Epidemiological studies in SSA based on a full night sleep monitoring with apnea-hypopnea index (AHI) measurement are very scarce. When performed, this measurement makes it possible to define the sleep-disordered breathing (SDB) as an AHI ≥ 5/h. Balkissou et al. found a SDB rate of 28.5% among an adult sample in Cameroon [13], while Benjafield et al.'s epidemiological estimation was 36.7% for Cameroon [16]. Besides those population-based data, hospital-based or clinical studies showed higher rates: 50%-77% for HR-OSAS in Nigeria and Cameroon [17–19] and 57.7% for SDB in Cameroon [20].

However, the 1999 and subsequent American Academy of Sleep Medicine (AASM) definitions of SAS include SDB (AHI ≥ 5/h) and either excessive daytime sleepiness, or one or more sleep respiratory event-related symptoms [21–23]. When such definitions are used, SAS prevalence in HIC drops to 3.1–7.5% in men and 1.2–4.5% in women [24–31]. To the best of our knowledge, only one epidemiological study assessed SAS using a 2-component definition and found a prevalence of 2.7% in adults in SSA [32]. On the other hand, we found no clinical study which used a 2-component definition to diagnose SAS.

Our study is aimed at determining among hospitalized patients, SAS prevalence using a variant of the 3^rd^ edition of The International Classification of Sleep Disorders (ICSD-3) definition, and describing the cooccurrence and association of SAS and some comorbidities.

## 2. Methods

### 2.1. Study Setting and Population

This cross-sectional study was conducted in Yaoundé Central Hospital (YCH) from November 2016 to May 2017. The study setting and population have been described in a previous paper from the same team [20].

### 2.2. Procedures

General procedure and data collection have been described in the aforementioned article [20]. In summary, patients were randomly enrolled in the global SAS study. Every day, 2 participants were randomly selected to be recorded by a portable sleep monitoring device (PMD), for SDB diagnosis (see below). For all participants, we collected the socio-demographic characteristics (age, sex, marital status, and occupational category), alcohol and tobacco consumption, and SAS-related symptoms. The physical examination included oropharynx assessment using the Mallampati score, which was dichotomized in low (score 1-2, corresponding to a good oropharynx opening) and high (score 3-4, having a poor oropharynx opening). The daytime sleepiness was also assessed using the Epwoth scale.

### 2.3. Comorbidity Assessment

Past or present history of hypertension, stroke, cardiac failure, diabetes mellitus, renal failure, epilepsy, and HIV infection was informed from the patient questioning or its medical file. All of these were presented as dichotomic (yes/no) variables. Obesity was defined as a body mass index (BMI) ≥ 30 kg/m^2^. Depression was screened using the 9-item Patient Health Questionnaire (PHQ9). This is a self-administered questionnaire that includes 9 questions coted 0 to 3 each, with increasing risk of having depression from 0 to 3. The minimum score is 0 and the maximum 27. We dichotomized the total score using the score 10 as threshold, to obtain either moderate to severe depression or not [33]. We used the self-administered Generalized Anxiety Disorders scale (GAD-7) to screen anxiety. This scale has 7 questions coted 0 to 3, and patients with a total score of 7 or more were suspected to have a significant anxiety disorder [34]. In addition to individual study, we grouped comorbidities in cardiovascular (at least one of hypertension or cardiac failure), global metabolic (obesity, diabetes mellitus, and renal failure), and neuropsychiatric or brain (stroke, epilepsy, moderate to severe depression, and significant anxiety disorder).

### 2.4. Sleep Apnea Syndrome Diagnosis

For SAS diagnosis, we considered the ICSD-3 definition, modifying it for more selectivity, since some authors found this definition too inclusive [35–37]. This included the combination of two simultaneous conditions: (1) the presence of SDB and (2) either excessive daytime sleepiness (based on complaint or appropriate assessment scale) or compatible symptoms (a minimum of 3 symptoms was required). Medical and psychiatric disorders were excluded from the definition, since many of them could be not related to SAS.

SDB was diagnosed using a portable sleep monitoring device (SLEEP FAIRY Lt, Co) as described by Poka-Mayap et al. [20]. The SDB could be obstructive (more than 50% of events are of obstructive) or central (more than 50% of events are of central type). An AHI of 5 or more events per hour was required, irrespective of their obstructive or central nature. The AHI threshold of 15 events/h was used to diagnose moderate to severe SDB (MS-SDB). The excessive daytime sleepiness was defined by either a complaint of sleepiness or an Epworth sleepiness scale (ESS) score > 10. Compatible symptoms consisted in the presence of at least 3 among these: severe and persisting snoring, breathless awakenings, nonrestoring sleep, daytime asthenia, impaired concentration or memory, and nocturia (more than one urination per night).

### 2.5. Data Management and Analysis

The required sample size calculation has been described elsewhere and was estimated 182 participants [20]. Data were analyzed using R software version 4.0.3 for Windows. Qualitative data were presented as counts and proportions. Quantitative variables were presented as mean ± standard deviation (SD) when they were normally distributed and as median (25^th^ percentile, 75^th^ percentile) otherwise. The normal distribution of continuous variables was assessed graphically. For each SAS feature, the prevalence was the proportion of patients who met the definition criteria among all of those with informed data on SDB and clinical criteria; it was presented with its 95% confident interval. Chi squared and Fisher tests were used to compare comorbidities rate with respect to the presence of SAS. A *p* value < 0.05 was used to define statistically significant results.

### 2.6. Ethics Statements

The study was approved by the Institutional Review Board of the Faculty of Medicine and Biomedical Sciences of the University of Yaoundé I, Cameroon (Clearance number 157/UY1/FMSB/VDRC/CSD of 24^th^ May 2017). The study received administrative authorization from the YCH administration (Reference number 024/AR/MINSANTE/SG/CHCY/AAMP of 20^th^ May 2017). Written informed consent was obtained from all participants.

## 3. Findings

### 3.1. Patient Characteristics

Of the 383 patients invited to participate in the study, 111 subjects ultimately had valid data including a sleep monitoring report. The flowchart of their inclusion is presented elsewhere [20]. The age of these 111 patients ranged 28 to 87 years, and more than half of them were female. Snoring (49.5%) and asthenia (85.6%) were the most common night and daytime symptoms, respectively. Daytime sleepiness was less frequent when diagnosed with ESS (9%) than as spontaneous patient complaint (64%). Two fifths of patients had a high Mallampati score, while the mean body mass index was 28.3 kg/m^2^. Patient characteristics are detailed on Table 1.

### 3.2. SAS Prevalence

Of the 111 patients who presented a valid sleep monitoring analysis, 71 were diagnosed with SDB and 61 met the diagnostic criteria of SAS. The related prevalences (95% CI) were 64.0% (55.0, 72.9) and 55.0% (45.7, 64.2), respectively. In the moderate to severe category, these rates dropped to 39.6% (30.5, 48.7) and 34.2% (25.4, 43.1), respectively (Table 2). The proportion of central SDB was low, but was higher in MS-SAS (13.1%) than in the overall group (8.9%). All patients with central SDB were symptomatic, and thus had a SAS (Table 2).

### 3.3. SAS and Comorbidities

**Figure 1:**
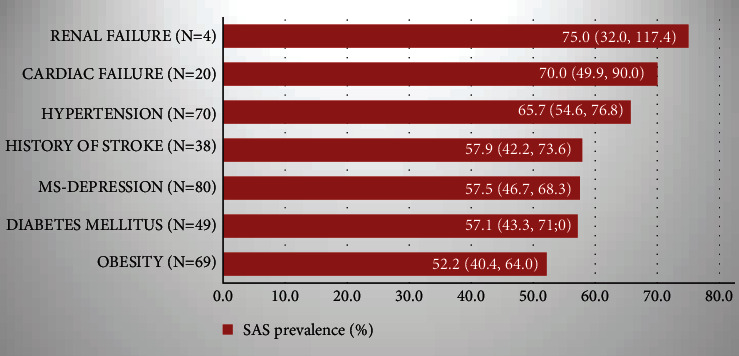
Prevalence of sleep apnea syndrome in patients with some comorbidities, hospitalized in Yaoundé Central Hospital, November 2016–May 2017, Yaoundé, Cameroun. *N* = 111. For each condition, SAS prevalence is given with its 95% confidence interval.

When considered as comorbidity of other conditions under study, the proportion of patients with SAS was high and varied according to the underlying diseases, from 52.2% in obese patients to 75.0% in kidney disease sufferers (Figure 1).

When SAS was considered as main disease, the comorbidities also varied widely. Among SAS patients, hypertension (75.4%) was more frequent than cardiac failure (23.0%). The presence of a cardiovascular condition (hypertension and/or cardiac failure) was significantly associated with SAS (*p* = 0.003) and MS-SAS (*p*˂0.001). Metabolic conditions (68.9%) were led by diabetes mellitus (45.9%), while the most frequent brain condition (44.3%) was stroke (36.7%). HIV infection was more frequent in SAS- patients (12.0%) than in SAS+ ones (3.3%). However, no significant association was found between SAS on one hand, and HIV infection, metabolic, or brain comorbidities on the other hand. These data are detailed on Table 3.

## 4. Discussion

In this study, realized among hospitalized patients with medical conditions, symptoms were dominated by asthenia and daytime sleepiness. The overall prevalence of SDB and that of SAS were high, most of the cases presenting obstructive SAS. The prevalence of SAS among participants with specific comorbidities ranged from 52.2% to 75.0%. All patients with central SDB ultimately had a significant SAS, while 85% of obstructive SDB showed SAS. Unlike metabolic and brain ones, cardiovascular (hypertension and cardiac failure) conditions were significantly more frequent in patients with SAS+ patients than in SAS-. Conversely, HIV infection appeared more frequent in SAS- patients than in SAS+.

Asthenia was likely overrepresented in our study, probably due to both preexisting and acute disease conditions that motivated the hospitalization. The large gap between Epworth-based and complaint-based sleepiness may be partly explained by difficulties in filling the Epworth questionnaire that could lead to an underestimation of the score in this Cameroonian population with a median age close to 60 years. These difficulties have been documented in the elderly nondemented Caucasian population [38, 39], as well as in a young healthy Cameroonian sample [40].

Globally, our SAS prevalence among patients suffering from cardiovascular (cardiac failure and hypertension) and cerebrovascular (stroke) comorbidities was consistent with published data from western settings [41–43]. The same trend could be observed for diabetes mellitus, as a recent review showed that 55 to 85% of diabetic patients have obstructive sleep apnea as comorbidity [44]. However, these were mainly epidemiological studies based on self-reported comorbidities. In our knowledge, clinical studies that assessed SAS prevalence in hospitalized patients are scarce, and those we found assessed SDB rather than SAS as defined in our study: Zhang et al. reported 66.7% of obstructive SDB among hospitalized diabetic patients in Beijing [45], while another study conducted on postmyocardial infarction subjects revealed a prevalence of 79% for the same disorder [46]. Those rates are higher than our prevalence for obstructive SDB (58.6%). This could be expected since our sample was less selected and less specific than those in the 2 studies. Notably, central SDB appeared to be more often significant (100% met the diagnosis of SAS vs. 84.5) and severe (83.3% were moderate to severe vs. 60.0%) than obstructive SDB. Despite the absence of comparative data regarding SAS prevalence among hospitalized patients with acute medical conditions, the rates we found are much higher than those described in epidemiological studies, usually ˂10%, supporting the association between SAS and these comorbidities. However, important differences not only in study populations but also in the criteria adopted to define SAS, between these epidemiological studies and our own, should be taken into consideration, when interpreting the results.

A discordant gap between SDB and SAS is noteworthy, when this clinical study is compared with epidemiological reports. In the same (Cameroonian) population, considering obstructive events, the SAS to SDB ratio (SAS prevalence/SDB prevalence) expressed in % could be estimated 7.5–9.6% in epidemiological studies [13, 16, 32], while it was 84.6% in the present study. This may suggest that comorbid patients are more often symptomatic regarding SDB; but it can also reflect an overestimation of OSAS prevalence, as some symptoms can be confounders (asthenia, impaired concentration or memory, daytime sleepiness, and nocturia).

As expected, hypertension was not only more frequent in SAS patients than others, but was also significantly associated with this condition in univariate analysis, as was global cardiovascular comorbidity. This association has been described in several studies in epidemiological western studies [4, 5, 47]. More recently in our setting, Njamnshi et al. reported 60% of hypertension in patients with HR-OSAS and only 20% in patients with no risk [19], while we found 75.4% in patients with confirmed SAS. Surprisingly, we found no association between SAS and metabolic comorbidities, and obesity and diabetes were even more frequent in non-MS-SAS than in MS-SAS. Recent systematic reviews and meta-analyses have shown a significant association between OSAS and metabolic syndrome [9], diabetes mellitus [44, 48], and renal failure [49]. We could hypothesize that HIV infection which is frequent among hospitalized patients partly contributed to these results, due to weight loss; but only 7.2% of patients were HIV+. The absence of a significant association between stroke and SAS was not expected in this study, since OSAS (which was the prevailing pattern) has been widely described as risk factor for stroke [50, 51]. The small number of patients in our sample, as well as the small proportion of some conditions (renal failure, stroke) could partly explain these differences. Our results concerning depression and SAS are consistent with the literature, showing no association between the two conditions, irrespective of the tool used to diagnose depression [52–54], although Rezaietalab et al. found high frequency of depressive (46.1%) and anxiety (53.9%) symptoms among 178 OSAS patients [55]. The absence of association between SAS and HIV infection appears to be in contrast with some studies from HIC [56–58] and a case-control study conducted in the same hospital (YCH) a few years ago [59]. This study showed that people living with HIV-AIDS (PLWHA) compared to controls had higher likelihood of OSA (43.6% versus 14.0%, adjusted odds ratio = 3.93, 95% confidence interval = 1.12–13.8) [59]. The study design (almost equal number of HIV+ and HIV- in the case-control study vs. only 7.2% of HIV+ in our study) could partly explain this discrepancy. Another explanatory factor could be the HIV+ profile: those described in previous studies were followed up under highly active antiretroviral therapy (HAART) for many years, and HAART regimen (especially when containing protease inhibitor) is known to cause lipodystrophy and obesity, associated with OSAS [57, 60].

To the best of our knowledge, this study was the first in SSA that assessed clinically significant SAS and its relationship with specific comorbidities, among hospitalized patients. The limitations of our study include the small sample size and use of portable monitory device rather than the gold standard (polysomnography) and have been largely discussed elsewhere [20].

## 5. Conclusion

Sleep apnea syndrome was frequent in this hospital-based study and was dominated by the obstructive pattern. This prevalence varied among patients with specific comorbidities, in consistency with current knowledge. Cardiovascular comorbidities were significantly more frequent in SAS patients, compared with SAS free ones, while metabolic and brain comorbidities showed no difference. This study supports the need to screen for comorbidities (especially cardiovascular) among SAS patients, and vice versa.

## Figures and Tables

**Table 1:** Sociodemographic, clinical, and sleep monitoring characteristics of patients hospitalized in Yaoundé Central Hospital, according to AHI category, November 2016–May 2017, Yaoundé, Cameroon.

Variables and categories	Overall (*N* = 111)	AHI < 5 (*N* = 37)	AHI ≥ 5 (*N* = 74)
Age (years)^∗^	58.0 ± 12.5 (28.0–87.0)	54.8 ± 13.3 (28.0–86.0)	59.6 ± 11.8 (32.0–87.0)
Female	59 (53.2)	17 (45.9)	42 (56.8)
Tobacco smoking	19 (17.1)	5 (13.5)	14 (18.9)
Alcohol consumption	18 (16.2)	5 (13.5)	13 (17.6)
Snoring	55 (49.5)	20 (54.1)	35 (47.3)
Breathless awakenings	19 (17.1)	3 (8.1)	16 (21.6)
Nocturia	33 (29.7)	11 (29.7)	22 (29.7)
Nonrestorative sleep	44 (39.6)	10 (27.0)	34 (45.9)
Morning headache	47 (42.3)	10 (27.0)	37 (50.0)
Daytime asthenia	95 (85.6)	30 (81.1)	65 (87.8)
Cognitive difficulties	53 (47.7)	16 (43.2)	37 (50.0)
Daytime sleepiness complaint	71 (64.0)	19 (51.4)	52 (70.3)
Epworth-based sleepiness	10 (9.0)	1 (2.7)	9 (12.2)
Epworth sleepiness scale^∗^	6.7 ± 3.2 (0.0–14.0)	6.2 ± 3.1 (0.0–12.0)	6.9 ± 3.2 (0.0–14.0)
Body mass index^∗^ (kg/m^2^)	28.3 ± 6.2 (16.4–50.8)	27.8 ± 6.0 (17.2–40.5)	28.6 ± 6.4 (16.4–50.8)
Mallampati score 3-4	45 (40.5)	10 (27.0)	35 (47.3)
Retrognatism	20 (18.0)	7 (18.9)	13 (17.6)
Apnea hypopnea index ^∗∗^ (events/h)	8.0 (1.9, 21.4), 0.0–119.5	1.0 (0.4, 1.8), 0.0–4.4	16.6 (7.4, 27.3), 5.0–119.5
Night O_2_ saturation^∗^ (%)	94.4 ± 3.0 (79.0–98.0)	95.8 ± 2.1 (88.0–98.0)	93.7 ± 3.2 (79.0–98.0)
Night desaturation index^∗∗^ (events/h)	13 (6.0, 29.0), 0.0–99.0	4 (2.0, 6.0), 0.0–99.0	22 (12.2, 36.7), 1.0–99.0

^∗^Mean ± standard deviation (range), ^∗∗^ median (1^st^–3^rd^ quartiles), range. Categorical data are expressed as number (frequency in %).

**Table 2:** Prevalence of sleep-disordered breathing and sleep apnea syndrome according to type and severity, among hospitalized patients in Yaoundé Central Hospital, November 2016–May 2017, Yaoundé, Cameroon. *N* = 111.

Sleep apnea syndromes features		Sleep-disordered breathing		Sleep apnea syndrome	
		Number	Prevalence (95% CI)^1^	Number	Prevalence (95% CI)^1^
Global	Overall	71	64.0 (55.0, 72.9)	61	55.0 (45.7, 64.2)
	Obstructive	65	58.6 (49.4, 67.7)	55	49.6 (40.2, 58.9)
	Central	6	5.4 (1.2, 9.6)	6	5.4 (1.2, 9.6)

Moderate to severe	Overall	44	39.6 (30.5, 48.7)	38	34.2 (25.4, 43.1)
	Obstructive	39	35.1 (26.3, 44.0)	33	29.7 2(21.2, 38.)
	Central	5	4.5 (0.6, 8.4)	5	4.5 (0.6, 8.4)

CI: confidence interval. ^1^Prevalence and confidence intervals are given in %.

**Table 3:** Frequencies of medical conditions (cardiovascular, metabolic, brain, and HIV infection) among patients with clinical sleep apnea syndrome, hospitalized in Yaoundé Central Hospital, November 2016–May 2017, Yaoundé, Cameroun. *N* = 111.

Medical conditions		Global sample	Overall SAS			Moderate to severe SAS		
			Yes (*N* = 61)	No (*N* = 50)	*p* value^∗^	Yes (*N* = 38)	No (*N* = 73)	*p* value^∗^
Cardiovascular	**Hypertension**	**70 (63.1)**	**46 (75.4)**	**24 (48.0)**	**0.005**	**32 (84.2)**	**38 (52.1)**	**0.002**
	Cardiac failure	20 (18.3)	14 (23.0)	6 (12.0)	0.213	9 (23.1)	11 (15.1)	0.390
	**Global cardiovascular**	**75 (67.6)**	**49 (80.3)**	**26 (52.0)**	**0.003**	**34 (89.5)**	**41 (52.2)**	**˂0.001**

Metabolic	Diabetes	49 (44.1)	28 (45.9)	21 (42.0)	0.826	14 (38.8)	35 (47.9)	0.359
	Obesity	42 (37.8)	25 (41.0)	17 (34.0)	0.577	14 (36.8)	28 (38.4)	1
	Renal failure	4 (3.6)	3 (4.9)	1 (2.0)	0.626	1 (2.6)	3 (4.1)	1
	**Global metabolic**	**71 (64.0)**	**42 (68.9)**	**29 (58.02)**	**0.324**	**22 (57.9)**	**49 (67.1)**	**0.452**

Brain	Stroke (*N* = 110)	38 (34.5)	22 (36.7)	16 (32.0)	0.756	15 (40.5)	23 (31.5)	0.466
	Moderate to severe depression (*N* = 101)	21 (20.8)	12 (20.7)	9 (20.9)	1.000	6 (17.1)	15 (22.7)	0.689
	Significant anxiety (*N* = 100)	1 (1.0)	1 (1.8)	0 (0.0)	1.000	1 (2.8)	0 (0.0)	0.360
	Epilepsy	5 (4.5)	2 (3.3)	3 (6.0)	0.656	2 (5.3)	3 (4.1)	1.000
	**Global brain**	**49 (44.1)**	**27 (44.3)**	**22 (44.0)**	**1.000**	**18 (47.4)**	**31 (42.5)**	**0.770**

HIV infection		**8 (7.2)**	**2 (3.3)**	**6 (12.0)**	**0.162**	**1 (2.6)**	**7 (9.6)**	**0.338**

Data are expressed as numbers (frequency in %). SAS: sleep apnea syndrome. ^∗^For comparison between groups, using Chi^2^, Chi^2^ with Yate's correction or Fisher test.

## Data Availability

The analysis file used to support the findings of this study are included within the supplementary information files and the dataset is available from the authors upon reasonable request.

## Supplementary Materials

Supplementary Materials The dataset used for our analysis and the output of our R analysis, generated using R markdown, are provided as csv and word files, respectively.
