# Supplementary material for: Sleep Apnea Syndrome: Prevalence and Comorbidity with Other Non-communicable Diseases and HIV Infection, among Hospitalized Patients in Yaoundé, Cameroon
Source: Sleep Disord. 2022 Feb 10;2022:4359294. doi: 10.1155/2022/4359294 (PMC8853793; doi:10.1155/2022/4359294)
Supplement: Supplementary Materials — The dataset used for our analysis and the output of our R analysis, generated using R markdown, are provided as csv and word files, respectively. [file 4359294.f1.doc]

# ANALYSES SAHS_ CV_HCY

MASSONGO MASSONGO

11/01/2022

## R Markdown

This is an R Markdown document. Markdown is a simple formatting syntax for authoring HTML, PDF, and MS Word documents. For more details on using R Markdown see [http://rmarkdown.rstudio.com](http://rmarkdown.rstudio.com/).

When you click the **Knit** button a document will be generated that includes both content as well as the output of any embedded R code chunks within the document. You can embed an R code chunk like this:

summary(cars)

## speed dist
## Min. : 4.0 Min. : 2.00
## 1st Qu.:12.0 1st Qu.: 26.00
## Median :15.0 Median : 36.00
## Mean :15.4 Mean : 42.98
## 3rd Qu.:19.0 3rd Qu.: 56.00
## Max. :25.0 Max. :120.00

## Including Plots

You can also embed plots, for example:


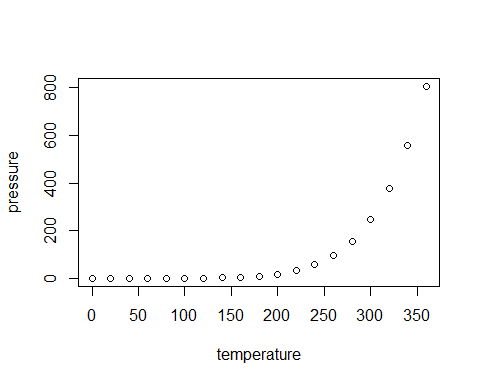


Note that the echo = FALSE parameter was added to the code chunk to prevent printing of the R code that generated the plot.

SAHS_HCY<-read.csv(file = "data_HCY.csv", sep = ";", dec = ".")
library(knitr)
library(questionr)
library(prettyR)

##
## Attaching package: 'prettyR'

## The following objects are masked from 'package:questionr':
##
## describe, freq

library(epiDisplay)

## Loading required package: foreign

## Loading required package: survival

## Loading required package: MASS

## Loading required package: nnet

library(stats)
library(childsds)

VARIABLE CRITERE A = IAHPOS

SAHS_HCY$IAHPOS=factor(SAHS_HCY$SAS,labels = c("1","0"), levels = c("1","2"))
 freq(SAHS_HCY$IAHPOS)

##
## Frequencies for SAHS_HCY$IAHPOS
## 1 0 NA
## 74 37 0
## % 66.7 33.3 0
## %!NA 66.7 33.3

Transformation symptomes SAHOS EN variables numériques

SAHS_HCY$nycturie[SAHS_HCY$NOMBRE=="1"]="0"
SAHS_HCY$nycturie[SAHS_HCY$NOMBRE=="2"]="1"
SAHS_HCY$nycturie=as.integer(SAHS_HCY$nycturie)
tabpct(SAHS_HCY$nycturie,SAHS_HCY$IAHPOS, percent = "col")

##
## Column percent
## SAHS_HCY$IAHPOS
## SAHS_HCY$nycturie 1 % 0 %
## 0 52 (70.3) 26 (70.3)
## 1 22 (29.7) 11 (29.7)
## Total 74 (100) 37 (100)


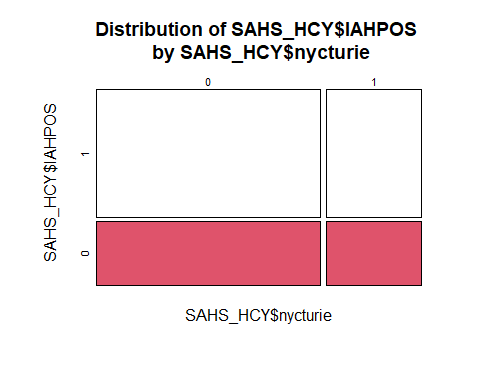


SAHS_HCY$ronfl = SAHS_HCY$RONFLEM1
tabpct(SAHS_HCY$ronfl,SAHS_HCY$IAHPOS, percent = "col")

##
## Column percent
## SAHS_HCY$IAHPOS
## SAHS_HCY$ronfl 1 % 0 %
## 0 39 (52.7) 17 (45.9)
## 1 35 (47.3) 20 (54.1)
## Total 74 (100) 37 (100)


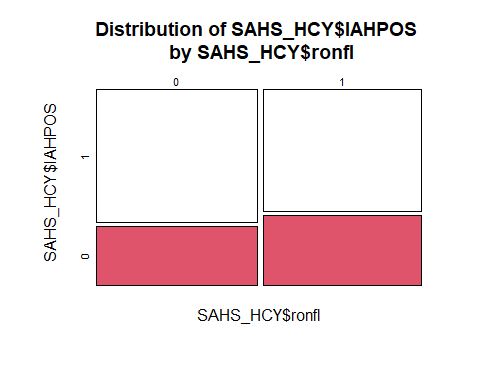


SAHS_HCY$suffoc = SAHS_HCY$RVEILS
SAHS_HCY$suffoc[SAHS_HCY$RVEILS=="2"]="0"
SAHS_HCY$suffoc=as.integer(SAHS_HCY$suffoc)
tabpct(SAHS_HCY$suffoc,SAHS_HCY$IAHPOS, percent = "col")

##
## Column percent
## SAHS_HCY$IAHPOS
## SAHS_HCY$suffoc 1 % 0 %
## 0 58 (78.4) 34 (91.9)
## 1 16 (21.6) 3 (8.1)
## Total 74 (100) 37 (100)


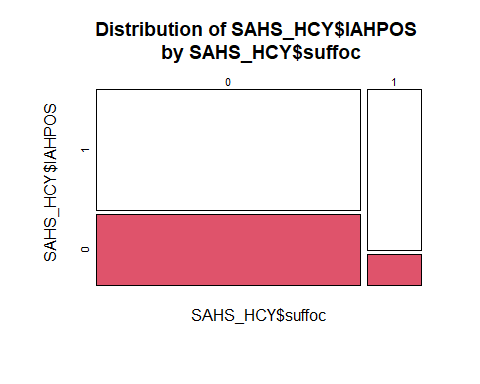


SAHS_HCY$SNR = SAHS_HCY$SOMNONRE
SAHS_HCY$SNR[SAHS_HCY$SOMNONRE=="2"]="0"
SAHS_HCY$SNR=as.integer(SAHS_HCY$SNR)
tabpct(SAHS_HCY$SNR,SAHS_HCY$IAHPOS, percent = "col")

##
## Column percent
## SAHS_HCY$IAHPOS
## SAHS_HCY$SNR 1 % 0 %
## 0 40 (54.1) 27 (73)
## 1 34 (45.9) 10 (27)
## Total 74 (100) 37 (100)


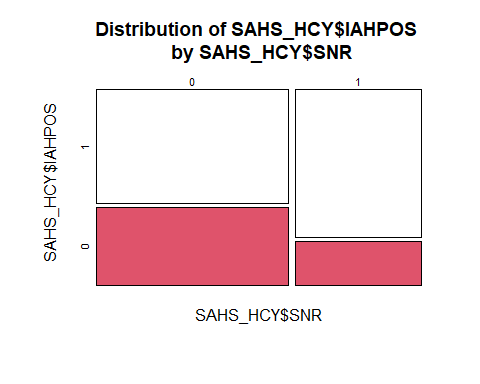


SAHS_HCY$asthenie = SAHS_HCY$FATIGABI
SAHS_HCY$asthenie[SAHS_HCY$FATIGABI=="2"]="0"
SAHS_HCY$asthenie=as.integer(SAHS_HCY$asthenie)
tabpct(SAHS_HCY$asthenie,SAHS_HCY$IAHPOS, percent = "col")

##
## Column percent
## SAHS_HCY$IAHPOS
## SAHS_HCY$asthenie 1 % 0 %
## 0 9 (12.2) 7 (18.9)
## 1 65 (87.8) 30 (81.1)
## Total 74 (100) 37 (100)


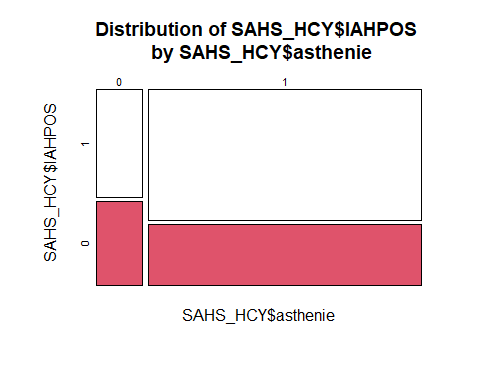


SAHS_HCY$tbconc = SAHS_HCY$TBLEMNSI
SAHS_HCY$tbconc[SAHS_HCY$TBLEMNSI=="2"]="0"
SAHS_HCY$tbconc=as.integer(SAHS_HCY$tbconc)
tabpct(SAHS_HCY$tbconc,SAHS_HCY$IAHPOS, percent = "col")

##
## Column percent
## SAHS_HCY$IAHPOS
## SAHS_HCY$tbconc 1 % 0 %
## 0 37 (50) 21 (56.8)
## 1 37 (50) 16 (43.2)
## Total 74 (100) 37 (100)


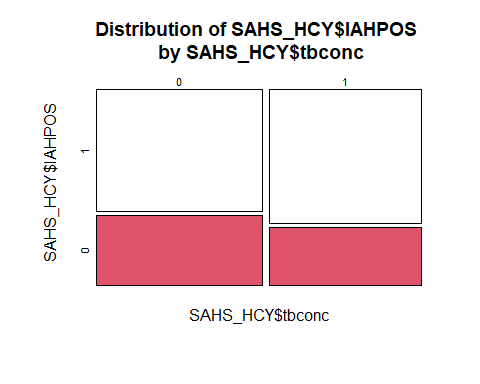
 CARACTERISTIQUES, COMPLEMENT

IMC

SAHS_HCY$BMI= SAHS_HCY$POIDS*10000/(SAHS_HCY$TAILLE*SAHS_HCY$TAILLE)
summary(SAHS_HCY$BMI)

## Min. 1st Qu. Median Mean 3rd Qu. Max.
## 16.36 24.36 27.78 28.33 31.40 50.78

sd(SAHS_HCY$BMI)

## [1] 6.245201

summary(SAHS_HCY$BMI[SAHS_HCY$IAHPOS=="0"])

## Min. 1st Qu. Median Mean 3rd Qu. Max.
## 17.21 23.44 27.68 27.81 30.49 40.46

sd(SAHS_HCY$BMI[SAHS_HCY$IAHPOS=="0"])

## [1] 5.993089

summary(SAHS_HCY$BMI[SAHS_HCY$IAHPOS=="1"])

## Min. 1st Qu. Median Mean 3rd Qu. Max.
## 16.36 24.72 27.98 28.59 31.62 50.78

sd(SAHS_HCY$BMI[SAHS_HCY$IAHPOS=="1"])

## [1] 6.391581

TABAGISME FUME

tabpct(SAHS_HCY$Tabagisme_Global,SAHS_HCY$IAHPOS, percent = "col", graph = "F")

##
## Column percent
## SAHS_HCY$IAHPOS
## SAHS_HCY$Tabagisme_Global 1 % 0 %
## 1 14 (18.9) 5 (13.5)
## 2 60 (81.1) 32 (86.5)
## Total 74 (100) 37 (100)

ETHYLISATION

tabpct(SAHS_HCY$Alcool_oui_non,SAHS_HCY$IAHPOS, percent = "col", graph = "F")

##
## Column percent
## SAHS_HCY$IAHPOS
## SAHS_HCY$Alcool_oui_non 1 % 0 %
## 1 13 (17.6) 5 (13.5)
## 2 61 (82.4) 32 (86.5)
## Total 74 (100) 37 (100)

SOMNOLEN

tabpct(SAHS_HCY$SOMNOLEN,SAHS_HCY$IAHPOS, percent = "col", graph = "F")

##
## Column percent
## SAHS_HCY$IAHPOS
## SAHS_HCY$SOMNOLEN 1 % 0 %
## 1 52 (70.3) 19 (51.4)
## 2 22 (29.7) 18 (48.6)
## Total 74 (100) 37 (100)

tabpct(SAHS_HCY$Somnolence_diurne,SAHS_HCY$IAHPOS, percent = "col", graph = "F")

##
## Column percent
## SAHS_HCY$IAHPOS
## SAHS_HCY$Somnolence_diurne 1 % 0 %
## 1 9 (12.2) 1 (2.7)
## 2 65 (87.8) 36 (97.3)
## Total 74 (100) 37 (100)

ESS

summary(SAHS_HCY$TOTEPWOR)

## Min. 1st Qu. Median Mean 3rd Qu. Max.
## 0.000 4.500 7.000 6.658 9.000 14.000

sd(SAHS_HCY$TOTEPWOR)

## [1] 3.169444

summary(SAHS_HCY$TOTEPWOR[SAHS_HCY$IAHPOS=="0"])

## Min. 1st Qu. Median Mean 3rd Qu. Max.
## 0.000 3.000 7.000 6.189 9.000 12.000

sd(SAHS_HCY$TOTEPWOR[SAHS_HCY$IAHPOS=="0"])

## [1] 3.098726

summary(SAHS_HCY$TOTEPWOR[SAHS_HCY$IAHPOS=="1"])

## Min. 1st Qu. Median Mean 3rd Qu. Max.
## 1.000 5.000 7.000 6.892 9.000 14.000

sd(SAHS_HCY$TOTEPWOR[SAHS_HCY$IAHPOS=="1"])

## [1] 3.199176

céphalée matinale

tabpct(SAHS_HCY$CPHALES,SAHS_HCY$IAHPOS, percent = "col", graph = "F")

##
## Column percent
## SAHS_HCY$IAHPOS
## SAHS_HCY$CPHALES 1 % 0 %
## 1 37 (50) 10 (27)
## 2 37 (50) 27 (73)
## Total 74 (100) 37 (100)

IAH

SAHS_HCY$IAH=SAHS_HCY$TOTEVTS*60/SAHS_HCY$TPSENREG
summary(SAHS_HCY$IAH)

## Min. 1st Qu. Median Mean 3rd Qu. Max.
## 0.000 1.909 7.087 14.994 21.363 119.468

sd(SAHS_HCY$IAH)

## [1] 18.34094

summary(SAHS_HCY$IAH[SAHS_HCY$IAHPOS=="0"])

## Min. 1st Qu. Median Mean 3rd Qu. Max.
## 0.0000 0.3909 0.9818 1.4018 1.7770 4.3617

sd(SAHS_HCY$IAH[SAHS_HCY$IAHPOS=="0"])

## [1] 1.361367

summary(SAHS_HCY$IAH[SAHS_HCY$IAHPOS=="1"])

## Min. 1st Qu. Median Mean 3rd Qu. Max.
## 3.153 7.409 16.563 21.790 27.293 119.468

sd(SAHS_HCY$IAH[SAHS_HCY$IAHPOS=="1"])

## [1] 19.11845

index desat

summary(SAHS_HCY$INDEXDES)

## Min. 1st Qu. Median Mean 3rd Qu. Max.
## 0.00 6.00 13.00 19.72 29.00 99.00

sd(SAHS_HCY$INDEXDES)

## [1] 18.74429

summary(SAHS_HCY$INDEXDES[SAHS_HCY$IAHPOS=="0"])

## Min. 1st Qu. Median Mean 3rd Qu. Max.
## 0.000 2.000 4.000 4.757 6.000 29.000

sd(SAHS_HCY$INDEXDES[SAHS_HCY$IAHPOS=="0"])

## [1] 4.884246

summary(SAHS_HCY$INDEXDES[SAHS_HCY$IAHPOS=="1"])

## Min. 1st Qu. Median Mean 3rd Qu. Max.
## 1.00 12.25 22.00 27.20 36.75 99.00

sd(SAHS_HCY$INDEXDES[SAHS_HCY$IAHPOS=="1"])

## [1] 18.63932

MALLAMPATI

summary(SAHS_HCY$MALLAMPA)

## Min. 1st Qu. Median Mean 3rd Qu. Max.
## 1.000 2.000 2.000 2.288 3.000 4.000

sd(SAHS_HCY$MALLAMPA)

## [1] 0.7907118

SAHS_HCY$mallampati=NA
SAHS_HCY$mallampati[SAHS_HCY$MALLAMPA=="1"|SAHS_HCY$MALLAMPA=="2"]="1-2"
SAHS_HCY$mallampati[SAHS_HCY$MALLAMPA=="3"|SAHS_HCY$MALLAMPA=="4"]="3-4"

tabpct(SAHS_HCY$mallampati,SAHS_HCY$IAHPOS, percent = "col", graph = "F")

##
## Column percent
## SAHS_HCY$IAHPOS
## SAHS_HCY$mallampati 1 % 0 %
## 1-2 39 (52.7) 27 (73)
## 3-4 35 (47.3) 10 (27)
## Total 74 (100) 37 (100)

RETROGNATISME

tab1(SAHS_HCY$RTROGNAT, graph = F)

## SAHS_HCY$RTROGNAT :
## Frequency Percent Cum. percent
## 1 20 18 18
## 2 91 82 100
## Total 111 100 100

tabpct(SAHS_HCY$RTROGNAT,SAHS_HCY$IAHPOS, percent = "col", graph = "F")

##
## Column percent
## SAHS_HCY$IAHPOS
## SAHS_HCY$RTROGNAT 1 % 0 %
## 1 13 (17.6) 7 (18.9)
## 2 61 (82.4) 30 (81.1)
## Total 74 (100) 37 (100)

SPO2

summary(SAHS_HCY$SPO2MOY)

## Min. 1st Qu. Median Mean 3rd Qu. Max.
## 79.00 93.00 95.00 94.41 96.00 98.00

sd(SAHS_HCY$SPO2MOY)

## [1] 3.01351

summary(SAHS_HCY$SPO2MOY[SAHS_HCY$IAHPOS=="0"])

## Min. 1st Qu. Median Mean 3rd Qu. Max.
## 88.00 95.00 96.00 95.81 97.00 98.00

sd(SAHS_HCY$SPO2MOY[SAHS_HCY$IAHPOS=="0"])

## [1] 2.079501

summary(SAHS_HCY$SPO2MOY[SAHS_HCY$IAHPOS=="1"])

## Min. 1st Qu. Median Mean 3rd Qu. Max.
## 79.00 93.00 94.00 93.72 96.00 98.00

sd(SAHS_HCY$SPO2MOY[SAHS_HCY$IAHPOS=="1"])

## [1] 3.173176

Variable somme symptomes significatifs. VARIABLE CRITERE C = SYMPT

SAHS_HCY$somsymp=SAHS_HCY$ronfl+SAHS_HCY$suffoc+SAHS_HCY$nycturie+SAHS_HCY$SNR+SAHS_HCY$asthenie+SAHS_HCY$tbconc
summary(SAHS_HCY$somsymp)

## Min. 1st Qu. Median Mean 3rd Qu. Max.
## 0.000 2.000 3.000 2.694 3.000 5.000

SAHS_HCY$SYMPT = cut(SAHS_HCY$somsymp,c(-1,2,6),labels = c("non","oui"))
tab1(SAHS_HCY$SYMPT, graph = F)

## SAHS_HCY$SYMPT :
## Frequency Percent Cum. percent
## non 44 39.6 39.6
## oui 67 60.4 100.0
## Total 111 100.0 100.0

SAHS_HCY$SYMPT_2 = cut(SAHS_HCY$somsymp,c(-1,1,6),labels = c("non","oui"))
tab1(SAHS_HCY$SYMPT_2, graph = F)

## SAHS_HCY$SYMPT_2 :
## Frequency Percent Cum. percent
## non 18 16.2 16.2
## oui 93 83.8 100.0
## Total 111 100.0 100.0

VARIABLE CRITERE B = SDE

SAHS_HCY$SDE<-NA
SAHS_HCY$SDE[SAHS_HCY$SOMNOLEN=="1"|SAHS_HCY$Somnolence_diurne=="1"]="oui"
SAHS_HCY$SDE[SAHS_HCY$SOMNOLEN=="2"& SAHS_HCY$Somnolence_diurne=="2"]="non"
table(SAHS_HCY$SDE)

##
## non oui
## 40 71

tab1(SAHS_HCY$SDE, graph = F)

## SAHS_HCY$SDE :
## Frequency Percent Cum. percent
## non 40 36 36
## oui 71 64 100
## Total 111 100 100

VARIABLE SYNDROME D’APNEES DE SOMMEIL = SASSIGN

SAHS_HCY$SASSIGN=NA
SAHS_HCY$SASSIGN[SAHS_HCY$IAHPOS=="1" & (SAHS_HCY$SDE=="oui"|SAHS_HCY$SYMPT=="oui")]="1"
SAHS_HCY$SASSIGN[SAHS_HCY$IAHPOS=="0" | SAHS_HCY$IAHPOS=="1" & SAHS_HCY$SDE=="non"& SAHS_HCY$SYMPT=="non"]="0"
SAHS_HCY$SASSIGN=as.factor(SAHS_HCY$SASSIGN)
tab1(SAHS_HCY$SASSIGN,graph = F)

## SAHS_HCY$SASSIGN :
## Frequency Percent Cum. percent
## 0 50 45 45
## 1 61 55 100
## Total 111 100 100

CARACTERISTIQUES POPULATION

summary(SAHS_HCY$AGE)

## Min. 1st Qu. Median Mean 3rd Qu. Max.
## 28.00 52.50 58.00 58.01 65.50 87.00

sd(SAHS_HCY$AGE)

## [1] 12.48526

summary(SAHS_HCY$AGE[SAHS_HCY$IAHPOS=="0"])

## Min. 1st Qu. Median Mean 3rd Qu. Max.
## 28.00 44.00 56.00 54.81 65.00 86.00

sd(SAHS_HCY$AGE[SAHS_HCY$IAHPOS=="0"])

## [1] 13.2829

summary(SAHS_HCY$AGE[SAHS_HCY$IAHPOS=="1"])

## Min. 1st Qu. Median Mean 3rd Qu. Max.
## 32.00 54.00 59.00 59.61 67.00 87.00

sd(SAHS_HCY$AGE[SAHS_HCY$IAHPOS=="1"])

## [1] 11.83658

tab1(SAHS_HCY$SEXE, graph = F)

## SAHS_HCY$SEXE :
## Frequency Percent Cum. percent
## 1 52 46.8 46.8
## 2 59 53.2 100.0
## Total 111 100.0 100.0

tabpct(SAHS_HCY$SEXE,SAHS_HCY$IAHPOS, percent = "col", graph = "F")

##
## Column percent
## SAHS_HCY$IAHPOS
## SAHS_HCY$SEXE 1 % 0 %
## 1 32 (43.2) 20 (54.1)
## 2 42 (56.8) 17 (45.9)
## Total 74 (100) 37 (100)

tab1(SAHS_HCY$IMC_4classes, graph = F)

## SAHS_HCY$IMC_4classes :
## Frequency Percent Cum. percent
## 1 11 9.9 9.9
## 2 26 23.4 33.3
## 3 32 28.8 62.2
## 4 42 37.8 100.0
## Total 111 100.0 100.0

tabpct(SAHS_HCY$IMC_4classes,SAHS_HCY$IAHPOS, percent = "col", graph = "F")

##
## Column percent
## SAHS_HCY$IAHPOS
## SAHS_HCY$IMC_4classes 1 % 0 %
## 1 7 (9.5) 4 (10.8)
## 2 14 (18.9) 12 (32.4)
## 3 25 (33.8) 7 (18.9)
## 4 28 (37.8) 14 (37.8)
## Total 74 (100) 37 (100)

comorbidité cardiovasculaire

tabpct(SAHS_HCY$SASSIGN,SAHS_HCY$HTA, graph = F)

##
## Original table
## SAHS_HCY$HTA
## SAHS_HCY$SASSIGN 1 2 Total
## 0 24 26 50
## 1 46 15 61
## Total 70 41 111
##
## Row percent
## SAHS_HCY$HTA
## SAHS_HCY$SASSIGN 1 2 Total
## 0 24 26 50
## (48) (52) (100)
## 1 46 15 61
## (75.4) (24.6) (100)
##
## Column percent
## SAHS_HCY$HTA
## SAHS_HCY$SASSIGN 1 % 2 %
## 0 24 (34.3) 26 (63.4)
## 1 46 (65.7) 15 (36.6)
## Total 70 (100) 41 (100)

chisq.test(SAHS_HCY$SASSIGN,SAHS_HCY$HTA, correct = TRUE)

##
## Pearson's Chi-squared test with Yates' continuity correction
##
## data: SAHS_HCY$SASSIGN and SAHS_HCY$HTA
## X-squared = 7.7248, df = 1, p-value = 0.005447

tabpct(SAHS_HCY$SASSIGN,SAHS_HCY$INSUFFCA, graph = F)

##
## Original table
## SAHS_HCY$INSUFFCA
## SAHS_HCY$SASSIGN 1 2 Total
## 0 6 44 50
## 1 14 47 61
## Total 20 91 111
##
## Row percent
## SAHS_HCY$INSUFFCA
## SAHS_HCY$SASSIGN 1 2 Total
## 0 6 44 50
## (12) (88) (100)
## 1 14 47 61
## (23) (77) (100)
##
## Column percent
## SAHS_HCY$INSUFFCA
## SAHS_HCY$SASSIGN 1 % 2 %
## 0 6 (30) 44 (48.4)
## 1 14 (70) 47 (51.6)
## Total 20 (100) 91 (100)

chisq.test(SAHS_HCY$SASSIGN,SAHS_HCY$INSUFFCA, correct = TRUE)

##
## Pearson's Chi-squared test with Yates' continuity correction
##
## data: SAHS_HCY$SASSIGN and SAHS_HCY$INSUFFCA
## X-squared = 1.551, df = 1, p-value = 0.213

CARDIOVACULAIRE GLOBAL

SAHS_HCY$CV="0"
SAHS_HCY$CV[SAHS_HCY$HTA=="1"|SAHS_HCY$INSUFFCA=="1"]="1"
SAHS_HCY$CV=as.factor(SAHS_HCY$CV)
tab1(SAHS_HCY$CV,graph = F)

## SAHS_HCY$CV :
## Frequency Percent Cum. percent
## 0 36 32.4 32.4
## 1 75 67.6 100.0
## Total 111 100.0 100.0

tabpct(SAHS_HCY$SASSIGN,SAHS_HCY$CV,graph = F)

##
## Original table
## SAHS_HCY$CV
## SAHS_HCY$SASSIGN 0 1 Total
## 0 24 26 50
## 1 12 49 61
## Total 36 75 111
##
## Row percent
## SAHS_HCY$CV
## SAHS_HCY$SASSIGN 0 1 Total
## 0 24 26 50
## (48) (52) (100)
## 1 12 49 61
## (19.7) (80.3) (100)
##
## Column percent
## SAHS_HCY$CV
## SAHS_HCY$SASSIGN 0 % 1 %
## 0 24 (66.7) 26 (34.7)
## 1 12 (33.3) 49 (65.3)
## Total 36 (100) 75 (100)

chisq.test(SAHS_HCY$SASSIGN,SAHS_HCY$CV, correct = TRUE)

##
## Pearson's Chi-squared test with Yates' continuity correction
##
## data: SAHS_HCY$SASSIGN and SAHS_HCY$CV
## X-squared = 8.8109, df = 1, p-value = 0.002994

COMORBIDITE METABOLIQUE

tabpct(SAHS_HCY$SASSIGN,SAHS_HCY$INSUFFRE, graph = F)

##
## Original table
## SAHS_HCY$INSUFFRE
## SAHS_HCY$SASSIGN 1 2 Total
## 0 1 49 50
## 1 3 58 61
## Total 4 107 111
##
## Row percent
## SAHS_HCY$INSUFFRE
## SAHS_HCY$SASSIGN 1 2 Total
## 0 1 49 50
## (2) (98) (100)
## 1 3 58 61
## (4.9) (95.1) (100)
##
## Column percent
## SAHS_HCY$INSUFFRE
## SAHS_HCY$SASSIGN 1 % 2 %
## 0 1 (25) 49 (45.8)
## 1 3 (75) 58 (54.2)
## Total 4 (100) 107 (100)

chisq.test(SAHS_HCY$SASSIGN,SAHS_HCY$INSUFFRE, correct = TRUE)

## Warning in chisq.test(SAHS_HCY$SASSIGN, SAHS_HCY$INSUFFRE, correct = TRUE): Chi-
## squared approximation may be incorrect

##
## Pearson's Chi-squared test with Yates' continuity correction
##
## data: SAHS_HCY$SASSIGN and SAHS_HCY$INSUFFRE
## X-squared = 0.095426, df = 1, p-value = 0.7574

fisher.test(SAHS_HCY$SASSIGN,SAHS_HCY$INSUFFRE)

##
## Fisher's Exact Test for Count Data
##
## data: SAHS_HCY$SASSIGN and SAHS_HCY$INSUFFRE
## p-value = 0.6257
## alternative hypothesis: true odds ratio is not equal to 1
## 95 percent confidence interval:
## 0.007373208 5.132749226
## sample estimates:
## odds ratio
## 0.3975836

SAHS_HCY$obesite=0
SAHS_HCY$obesite[SAHS_HCY$Tranche_IMC1=="4"|SAHS_HCY$Tranche_IMC1=="5"|SAHS_HCY$Tranche_IMC1=="6"]="1"
SAHS_HCY$obesite=as.factor(SAHS_HCY$obesite)
table(SAHS_HCY$obesite)

##
## 0 1
## 69 42

tabpct(SAHS_HCY$SASSIGN,SAHS_HCY$obesite, graph = F)

##
## Original table
## SAHS_HCY$obesite
## SAHS_HCY$SASSIGN 0 1 Total
## 0 33 17 50
## 1 36 25 61
## Total 69 42 111
##
## Row percent
## SAHS_HCY$obesite
## SAHS_HCY$SASSIGN 0 1 Total
## 0 33 17 50
## (66) (34) (100)
## 1 36 25 61
## (59) (41) (100)
##
## Column percent
## SAHS_HCY$obesite
## SAHS_HCY$SASSIGN 0 % 1 %
## 0 33 (47.8) 17 (40.5)
## 1 36 (52.2) 25 (59.5)
## Total 69 (100) 42 (100)

chisq.test(SAHS_HCY$SASSIGN,SAHS_HCY$obesite, correct = TRUE)

##
## Pearson's Chi-squared test with Yates' continuity correction
##
## data: SAHS_HCY$SASSIGN and SAHS_HCY$obesite
## X-squared = 0.31152, df = 1, p-value = 0.5767

tabpct(SAHS_HCY$SASSIGN,SAHS_HCY$DIABTE, graph = F)

##
## Original table
## SAHS_HCY$DIABTE
## SAHS_HCY$SASSIGN 1 2 Total
## 0 21 29 50
## 1 28 33 61
## Total 49 62 111
##
## Row percent
## SAHS_HCY$DIABTE
## SAHS_HCY$SASSIGN 1 2 Total
## 0 21 29 50
## (42) (58) (100)
## 1 28 33 61
## (45.9) (54.1) (100)
##
## Column percent
## SAHS_HCY$DIABTE
## SAHS_HCY$SASSIGN 1 % 2 %
## 0 21 (42.9) 29 (46.8)
## 1 28 (57.1) 33 (53.2)
## Total 49 (100) 62 (100)

chisq.test(SAHS_HCY$SASSIGN,SAHS_HCY$DIABTE, correct = TRUE)

##
## Pearson's Chi-squared test with Yates' continuity correction
##
## data: SAHS_HCY$SASSIGN and SAHS_HCY$DIABTE
## X-squared = 0.048304, df = 1, p-value = 0.826

METABOLIQUE GLOBAL

SAHS_HCY$METAB="0"
SAHS_HCY$METAB[SAHS_HCY$DIABTE=="1"|SAHS_HCY$INSUFFRE=="1"|SAHS_HCY$obesite=="1"]="1"
SAHS_HCY$METAB=as.factor(SAHS_HCY$METAB)
tab1(SAHS_HCY$METAB,graph = F)

## SAHS_HCY$METAB :
## Frequency Percent Cum. percent
## 0 40 36 36
## 1 71 64 100
## Total 111 100 100

tabpct(SAHS_HCY$SASSIGN,SAHS_HCY$METAB,graph = F)

##
## Original table
## SAHS_HCY$METAB
## SAHS_HCY$SASSIGN 0 1 Total
## 0 21 29 50
## 1 19 42 61
## Total 40 71 111
##
## Row percent
## SAHS_HCY$METAB
## SAHS_HCY$SASSIGN 0 1 Total
## 0 21 29 50
## (42) (58) (100)
## 1 19 42 61
## (31.1) (68.9) (100)
##
## Column percent
## SAHS_HCY$METAB
## SAHS_HCY$SASSIGN 0 % 1 %
## 0 21 (52.5) 29 (40.8)
## 1 19 (47.5) 42 (59.2)
## Total 40 (100) 71 (100)

chisq.test(SAHS_HCY$SASSIGN,SAHS_HCY$METAB, correct = TRUE)

##
## Pearson's Chi-squared test with Yates' continuity correction
##
## data: SAHS_HCY$SASSIGN and SAHS_HCY$METAB
## X-squared = 0.97263, df = 1, p-value = 0.324

COMORBIDITE NEURO PSYCHIATRIQUE (BRAIN)

RECODAGE PSYCHIATRIQUE

SAHS_HCY$depr_MS=cut(SAHS_HCY$TOTALPHQ,c(0,9,30),labels = c("non","oui"))
table(SAHS_HCY$depr_MS)

##
## non oui
## 80 21

SAHS_HCY$anxiete=cut(SAHS_HCY$TOTALGAD,c(0,14,40),labels = c("non","oui"))
table(SAHS_HCY$anxiete)

##
## non oui
## 99 1

PSYCHIATRIQUE

SAHS_HCY$depr_MS=as.factor(SAHS_HCY$depr_MS)
tabpct(SAHS_HCY$SASSIGN,SAHS_HCY$depr_MS,graph = F)

##
## Original table
## SAHS_HCY$depr_MS
## SAHS_HCY$SASSIGN non oui Total
## 0 34 9 43
## 1 46 12 58
## Total 80 21 101
##
## Row percent
## SAHS_HCY$depr_MS
## SAHS_HCY$SASSIGN non oui Total
## 0 34 9 43
## (79.1) (20.9) (100)
## 1 46 12 58
## (79.3) (20.7) (100)
##
## Column percent
## SAHS_HCY$depr_MS
## SAHS_HCY$SASSIGN non % oui %
## 0 34 (42.5) 9 (42.9)
## 1 46 (57.5) 12 (57.1)
## Total 80 (100) 21 (100)

chisq.test(SAHS_HCY$SASSIGN,SAHS_HCY$depr_MS, correct = TRUE)

##
## Pearson's Chi-squared test with Yates' continuity correction
##
## data: SAHS_HCY$SASSIGN and SAHS_HCY$depr_MS
## X-squared = 1.6133e-31, df = 1, p-value = 1

SAHS_HCY$anxiete=as.factor(SAHS_HCY$anxiete)
tabpct(SAHS_HCY$SASSIGN,SAHS_HCY$anxiete,graph = F)

##
## Original table
## SAHS_HCY$anxiete
## SAHS_HCY$SASSIGN non oui Total
## 0 44 0 44
## 1 55 1 56
## Total 99 1 100
##
## Row percent
## SAHS_HCY$anxiete
## SAHS_HCY$SASSIGN non oui Total
## 0 44 0 44
## (100) (0) (100)
## 1 55 1 56
## (98.2) (1.8) (100)
##
## Column percent
## SAHS_HCY$anxiete
## SAHS_HCY$SASSIGN non % oui %
## 0 44 (44.4) 0 (0)
## 1 55 (55.6) 1 (100)
## Total 99 (100) 1 (100)

chisq.test(SAHS_HCY$SASSIGN,SAHS_HCY$anxiete, correct = TRUE)

## Warning in chisq.test(SAHS_HCY$SASSIGN, SAHS_HCY$anxiete, correct = TRUE): Chi-
## squared approximation may be incorrect

##
## Pearson's Chi-squared test with Yates' continuity correction
##
## data: SAHS_HCY$SASSIGN and SAHS_HCY$anxiete
## X-squared = 2.0577e-29, df = 1, p-value = 1

AVC

tabpct(SAHS_HCY$SASSIGN,SAHS_HCY$AVC, graph = F)

##
## Original table
## SAHS_HCY$AVC
## SAHS_HCY$SASSIGN 1 2 Total
## 0 16 34 50
## 1 22 38 60
## Total 38 72 110
##
## Row percent
## SAHS_HCY$AVC
## SAHS_HCY$SASSIGN 1 2 Total
## 0 16 34 50
## (32) (68) (100)
## 1 22 38 60
## (36.7) (63.3) (100)
##
## Column percent
## SAHS_HCY$AVC
## SAHS_HCY$SASSIGN 1 % 2 %
## 0 16 (42.1) 34 (47.2)
## 1 22 (57.9) 38 (52.8)
## Total 38 (100) 72 (100)

chisq.test(SAHS_HCY$SASSIGN,SAHS_HCY$AVC, correct = TRUE)

##
## Pearson's Chi-squared test with Yates' continuity correction
##
## data: SAHS_HCY$SASSIGN and SAHS_HCY$AVC
## X-squared = 0.096826, df = 1, p-value = 0.7557

EPILEPSIE

SAHS_HCY$EPILEPSI=as.factor(SAHS_HCY$EPILEPSI)
tabpct(SAHS_HCY$SASSIGN,SAHS_HCY$EPILEPSI,graph = F)

##
## Original table
## SAHS_HCY$EPILEPSI
## SAHS_HCY$SASSIGN 1 2 Total
## 0 3 47 50
## 1 2 59 61
## Total 5 106 111
##
## Row percent
## SAHS_HCY$EPILEPSI
## SAHS_HCY$SASSIGN 1 2 Total
## 0 3 47 50
## (6) (94) (100)
## 1 2 59 61
## (3.3) (96.7) (100)
##
## Column percent
## SAHS_HCY$EPILEPSI
## SAHS_HCY$SASSIGN 1 % 2 %
## 0 3 (60) 47 (44.3)
## 1 2 (40) 59 (55.7)
## Total 5 (100) 106 (100)

chisq.test(SAHS_HCY$SASSIGN,SAHS_HCY$EPILEPSI, correct = TRUE)

## Warning in chisq.test(SAHS_HCY$SASSIGN, SAHS_HCY$EPILEPSI, correct = TRUE): Chi-
## squared approximation may be incorrect

##
## Pearson's Chi-squared test with Yates' continuity correction
##
## data: SAHS_HCY$SASSIGN and SAHS_HCY$EPILEPSI
## X-squared = 0.051929, df = 1, p-value = 0.8197

fisher.test(SAHS_HCY$SASSIGN,SAHS_HCY$EPILEPSI)

##
## Fisher's Exact Test for Count Data
##
## data: SAHS_HCY$SASSIGN and SAHS_HCY$EPILEPSI
## p-value = 0.656
## alternative hypothesis: true odds ratio is not equal to 1
## 95 percent confidence interval:
## 0.2056679 23.2711362
## sample estimates:
## odds ratio
## 1.872206

GLOBAL BRAIN

SAHS_HCY$NEUROPSY="0"
SAHS_HCY$NEUROPSY[SAHS_HCY$depr_MS=="oui"|SAHS_HCY$anxiete=="oui"|SAHS_HCY$EPILEPSI=="1"|SAHS_HCY$AVC=="1"]="1"
SAHS_HCY$NEUROPSY=as.factor(SAHS_HCY$NEUROPSY)
tab1(SAHS_HCY$NEUROPSY,graph = F)

## SAHS_HCY$NEUROPSY :
## Frequency Percent Cum. percent
## 0 62 55.9 55.9
## 1 49 44.1 100.0
## Total 111 100.0 100.0

tabpct(SAHS_HCY$SASSIGN,SAHS_HCY$NEUROPSY,graph = F)

##
## Original table
## SAHS_HCY$NEUROPSY
## SAHS_HCY$SASSIGN 0 1 Total
## 0 28 22 50
## 1 34 27 61
## Total 62 49 111
##
## Row percent
## SAHS_HCY$NEUROPSY
## SAHS_HCY$SASSIGN 0 1 Total
## 0 28 22 50
## (56) (44) (100)
## 1 34 27 61
## (55.7) (44.3) (100)
##
## Column percent
## SAHS_HCY$NEUROPSY
## SAHS_HCY$SASSIGN 0 % 1 %
## 0 28 (45.2) 22 (44.9)
## 1 34 (54.8) 27 (55.1)
## Total 62 (100) 49 (100)

chisq.test(SAHS_HCY$SASSIGN,SAHS_HCY$NEUROPSY, correct = TRUE)

##
## Pearson's Chi-squared test with Yates' continuity correction
##
## data: SAHS_HCY$SASSIGN and SAHS_HCY$NEUROPSY
## X-squared = 3.7044e-31, df = 1, p-value = 1

fisher.test(SAHS_HCY$SASSIGN,SAHS_HCY$NEUROPSY)

##
## Fisher's Exact Test for Count Data
##
## data: SAHS_HCY$SASSIGN and SAHS_HCY$NEUROPSY
## p-value = 1
## alternative hypothesis: true odds ratio is not equal to 1
## 95 percent confidence interval:
## 0.4453745 2.2991586
## sample estimates:
## odds ratio
## 1.0106

VIH

SAHS_HCY$VIH=as.factor(SAHS_HCY$VIH)
tabpct(SAHS_HCY$SASSIGN,SAHS_HCY$VIH,graph = F)

##
## Original table
## SAHS_HCY$VIH
## SAHS_HCY$SASSIGN 1 2 Total
## 0 6 44 50
## 1 2 59 61
## Total 8 103 111
##
## Row percent
## SAHS_HCY$VIH
## SAHS_HCY$SASSIGN 1 2 Total
## 0 6 44 50
## (12) (88) (100)
## 1 2 59 61
## (3.3) (96.7) (100)
##
## Column percent
## SAHS_HCY$VIH
## SAHS_HCY$SASSIGN 1 % 2 %
## 0 6 (75) 44 (42.7)
## 1 2 (25) 59 (57.3)
## Total 8 (100) 103 (100)

chisq.test(SAHS_HCY$SASSIGN,SAHS_HCY$VIH, correct = TRUE)

## Warning in chisq.test(SAHS_HCY$SASSIGN, SAHS_HCY$VIH, correct = TRUE): Chi-
## squared approximation may be incorrect

##
## Pearson's Chi-squared test with Yates' continuity correction
##
## data: SAHS_HCY$SASSIGN and SAHS_HCY$VIH
## X-squared = 1.957, df = 1, p-value = 0.1618

fisher.test(SAHS_HCY$SASSIGN,SAHS_HCY$VIH)

##
## Fisher's Exact Test for Count Data
##
## data: SAHS_HCY$SASSIGN and SAHS_HCY$VIH
## p-value = 0.137
## alternative hypothesis: true odds ratio is not equal to 1
## 95 percent confidence interval:
## 0.6697161 42.1397215
## sample estimates:
## odds ratio
## 3.974502

INTRODUCTION IAH

SAHS_BIS<-read.csv(file = "data_SAHS_HCY_app_2.csv", sep = ";", dec = ".")

SAHS_NEW=merge(SAHS_HCY,SAHS_BIS[,c("NUMEROFI","IAH")],by="NUMEROFI")
SAHS_NEW$IAH.y=as.numeric(SAHS_NEW$IAH.y)
summary(SAHS_NEW$IAH.y)

## Min. 1st Qu. Median Mean 3rd Qu. Max.
## 0.00 2.00 8.00 15.32 22.00 120.00

Variable IAH MODERE A SEVERE

SAHS_NEW$IAH_MS=cut(SAHS_NEW$IAH.y,c("-1","14.9999","200"),labels = c("0","1"))

tab1(SAHS_NEW$IAH_MS,graph = F)

## SAHS_NEW$IAH_MS :
## Frequency Percent Cum. percent
## 0 67 60.4 60.4
## 1 44 39.6 100.0
## Total 111 100.0 100.0

VARIABLE SAHSMS SIGN

SAHS_NEW$SAHSMS=NA
SAHS_NEW$SAHSMS[SAHS_NEW$IAH_MS=="1" & (SAHS_NEW$SDE=="oui"|SAHS_NEW$SYMPT=="oui")]="1"
SAHS_NEW$SAHSMS[SAHS_NEW$IAH_MS=="0" | SAHS_NEW$IAH_MS=="1" & SAHS_NEW$SDE=="non"& SAHS_NEW$SYMPT=="non"]="0"
SAHS_NEW$SAHSMS=as.factor(SAHS_NEW$SAHSMS)
tab1(SAHS_NEW$SAHSMS,graph = F)

## SAHS_NEW$SAHSMS :
## Frequency Percent Cum. percent
## 0 73 65.8 65.8
## 1 38 34.2 100.0
## Total 111 100.0 100.0

comorbidité cardiovasculaire

tabpct(SAHS_NEW$SAHSMS,SAHS_NEW$HTA, graph = F)

##
## Original table
## SAHS_NEW$HTA
## SAHS_NEW$SAHSMS 1 2 Total
## 0 38 35 73
## 1 32 6 38
## Total 70 41 111
##
## Row percent
## SAHS_NEW$HTA
## SAHS_NEW$SAHSMS 1 2 Total
## 0 38 35 73
## (52.1) (47.9) (100)
## 1 32 6 38
## (84.2) (15.8) (100)
##
## Column percent
## SAHS_NEW$HTA
## SAHS_NEW$SAHSMS 1 % 2 %
## 0 38 (54.3) 35 (85.4)
## 1 32 (45.7) 6 (14.6)
## Total 70 (100) 41 (100)

chisq.test(SAHS_NEW$SAHSMS,SAHS_NEW$HTA, correct = TRUE)

##
## Pearson's Chi-squared test with Yates' continuity correction
##
## data: SAHS_NEW$SAHSMS and SAHS_NEW$HTA
## X-squared = 9.7559, df = 1, p-value = 0.001787

tabpct(SAHS_NEW$SAHSMS,SAHS_NEW$INSUFFCA, graph = F)

##
## Original table
## SAHS_NEW$INSUFFCA
## SAHS_NEW$SAHSMS 1 2 Total
## 0 11 62 73
## 1 9 29 38
## Total 20 91 111
##
## Row percent
## SAHS_NEW$INSUFFCA
## SAHS_NEW$SAHSMS 1 2 Total
## 0 11 62 73
## (15.1) (84.9) (100)
## 1 9 29 38
## (23.7) (76.3) (100)
##
## Column percent
## SAHS_NEW$INSUFFCA
## SAHS_NEW$SAHSMS 1 % 2 %
## 0 11 (55) 62 (68.1)
## 1 9 (45) 29 (31.9)
## Total 20 (100) 91 (100)

chisq.test(SAHS_NEW$SAHSMS,SAHS_NEW$INSUFFCA, correct = TRUE)

##
## Pearson's Chi-squared test with Yates' continuity correction
##
## data: SAHS_NEW$SAHSMS and SAHS_NEW$INSUFFCA
## X-squared = 0.74032, df = 1, p-value = 0.3896

CARDIOVACULAIRE GLOBAL

SAHS_NEW$CV="0"
SAHS_NEW$CV[SAHS_NEW$HTA=="1"|SAHS_NEW$INSUFFCA=="1"]="1"
SAHS_NEW$CV=as.factor(SAHS_NEW$CV)
tab1(SAHS_NEW$CV,graph = F)

## SAHS_NEW$CV :
## Frequency Percent Cum. percent
## 0 36 32.4 32.4
## 1 75 67.6 100.0
## Total 111 100.0 100.0

tabpct(SAHS_NEW$SAHSMS,SAHS_NEW$CV,graph = F)

##
## Original table
## SAHS_NEW$CV
## SAHS_NEW$SAHSMS 0 1 Total
## 0 32 41 73
## 1 4 34 38
## Total 36 75 111
##
## Row percent
## SAHS_NEW$CV
## SAHS_NEW$SAHSMS 0 1 Total
## 0 32 41 73
## (43.8) (56.2) (100)
## 1 4 34 38
## (10.5) (89.5) (100)
##
## Column percent
## SAHS_NEW$CV
## SAHS_NEW$SAHSMS 0 % 1 %
## 0 32 (88.9) 41 (54.7)
## 1 4 (11.1) 34 (45.3)
## Total 36 (100) 75 (100)

chisq.test(SAHS_NEW$SAHSMS,SAHS_NEW$CV, correct = TRUE)

##
## Pearson's Chi-squared test with Yates' continuity correction
##
## data: SAHS_NEW$SAHSMS and SAHS_NEW$CV
## X-squared = 11.179, df = 1, p-value = 0.0008274

COMORBIDITE METABOLIQUE

tabpct(SAHS_NEW$SAHSMS,SAHS_NEW$INSUFFRE, graph = F)

##
## Original table
## SAHS_NEW$INSUFFRE
## SAHS_NEW$SAHSMS 1 2 Total
## 0 3 70 73
## 1 1 37 38
## Total 4 107 111
##
## Row percent
## SAHS_NEW$INSUFFRE
## SAHS_NEW$SAHSMS 1 2 Total
## 0 3 70 73
## (4.1) (95.9) (100)
## 1 1 37 38
## (2.6) (97.4) (100)
##
## Column percent
## SAHS_NEW$INSUFFRE
## SAHS_NEW$SAHSMS 1 % 2 %
## 0 3 (75) 70 (65.4)
## 1 1 (25) 37 (34.6)
## Total 4 (100) 107 (100)

chisq.test(SAHS_NEW$SAHSMS,SAHS_NEW$INSUFFRE, correct = TRUE)

## Warning in chisq.test(SAHS_NEW$SAHSMS, SAHS_NEW$INSUFFRE, correct = TRUE): Chi-
## squared approximation may be incorrect

##
## Pearson's Chi-squared test with Yates' continuity correction
##
## data: SAHS_NEW$SAHSMS and SAHS_NEW$INSUFFRE
## X-squared = 1.1775e-29, df = 1, p-value = 1

fisher.test(SAHS_NEW$SAHSMS,SAHS_NEW$INSUFFRE)

##
## Fisher's Exact Test for Count Data
##
## data: SAHS_NEW$SAHSMS and SAHS_NEW$INSUFFRE
## p-value = 1
## alternative hypothesis: true odds ratio is not equal to 1
## 95 percent confidence interval:
## 0.1218974 85.4274982
## sample estimates:
## odds ratio
## 1.579644

SAHS_NEW$obesite=0
SAHS_NEW$obesite[SAHS_NEW$Tranche_IMC1=="4"|SAHS_NEW$Tranche_IMC1=="5"|SAHS_NEW$Tranche_IMC1=="6"]="1"
SAHS_NEW$obesite=as.factor(SAHS_NEW$obesite)
table(SAHS_NEW$obesite)

##
## 0 1
## 69 42

tabpct(SAHS_NEW$SAHSMS,SAHS_NEW$obesite, graph = F)

##
## Original table
## SAHS_NEW$obesite
## SAHS_NEW$SAHSMS 0 1 Total
## 0 45 28 73
## 1 24 14 38
## Total 69 42 111
##
## Row percent
## SAHS_NEW$obesite
## SAHS_NEW$SAHSMS 0 1 Total
## 0 45 28 73
## (61.6) (38.4) (100)
## 1 24 14 38
## (63.2) (36.8) (100)
##
## Column percent
## SAHS_NEW$obesite
## SAHS_NEW$SAHSMS 0 % 1 %
## 0 45 (65.2) 28 (66.7)
## 1 24 (34.8) 14 (33.3)
## Total 69 (100) 42 (100)

chisq.test(SAHS_NEW$SAHSMS,SAHS_NEW$obesite, correct = TRUE)

##
## Pearson's Chi-squared test with Yates' continuity correction
##
## data: SAHS_NEW$SAHSMS and SAHS_NEW$obesite
## X-squared = 0, df = 1, p-value = 1

tabpct(SAHS_NEW$SAHSMS,SAHS_NEW$DIABTE, graph = F)

##
## Original table
## SAHS_NEW$DIABTE
## SAHS_NEW$SAHSMS 1 2 Total
## 0 35 38 73
## 1 14 24 38
## Total 49 62 111
##
## Row percent
## SAHS_NEW$DIABTE
## SAHS_NEW$SAHSMS 1 2 Total
## 0 35 38 73
## (47.9) (52.1) (100)
## 1 14 24 38
## (36.8) (63.2) (100)
##
## Column percent
## SAHS_NEW$DIABTE
## SAHS_NEW$SAHSMS 1 % 2 %
## 0 35 (71.4) 38 (61.3)
## 1 14 (28.6) 24 (38.7)
## Total 49 (100) 62 (100)

chisq.test(SAHS_NEW$SAHSMS,SAHS_NEW$DIABTE, correct = TRUE)

##
## Pearson's Chi-squared test with Yates' continuity correction
##
## data: SAHS_NEW$SAHSMS and SAHS_NEW$DIABTE
## X-squared = 0.83975, df = 1, p-value = 0.3595

METABOLIQUE GLOBAL

SAHS_NEW$METAB="0"
SAHS_NEW$METAB[SAHS_NEW$DIABTE=="1"|SAHS_NEW$INSUFFRE=="1"|SAHS_NEW$obesite=="1"]="1"
SAHS_NEW$METAB=as.factor(SAHS_NEW$METAB)
tab1(SAHS_NEW$METAB,graph = F)

## SAHS_NEW$METAB :
## Frequency Percent Cum. percent
## 0 40 36 36
## 1 71 64 100
## Total 111 100 100

tabpct(SAHS_NEW$SAHSMS,SAHS_NEW$METAB,graph = F)

##
## Original table
## SAHS_NEW$METAB
## SAHS_NEW$SAHSMS 0 1 Total
## 0 24 49 73
## 1 16 22 38
## Total 40 71 111
##
## Row percent
## SAHS_NEW$METAB
## SAHS_NEW$SAHSMS 0 1 Total
## 0 24 49 73
## (32.9) (67.1) (100)
## 1 16 22 38
## (42.1) (57.9) (100)
##
## Column percent
## SAHS_NEW$METAB
## SAHS_NEW$SAHSMS 0 % 1 %
## 0 24 (60) 49 (69)
## 1 16 (40) 22 (31)
## Total 40 (100) 71 (100)

chisq.test(SAHS_NEW$SAHSMS,SAHS_NEW$METAB, correct = TRUE)

##
## Pearson's Chi-squared test with Yates' continuity correction
##
## data: SAHS_NEW$SAHSMS and SAHS_NEW$METAB
## X-squared = 0.5664, df = 1, p-value = 0.4517

COMORBIDITE NEURO PSYCHIATRIQUE

SAHS_NEW$depr_MS=cut(SAHS_NEW$TOTALPHQ,c(0,9,30),labels = c("non","oui"))
table(SAHS_NEW$depr_MS)

##
## non oui
## 80 21

SAHS_NEW$anxiete=cut(SAHS_NEW$TOTALGAD,c(0,14,40),labels = c("non","oui"))
table(SAHS_NEW$anxiete)

##
## non oui
## 99 1

SAHS_NEW$depr_MS=as.factor(SAHS_NEW$depr_MS)
tabpct(SAHS_NEW$SAHSMS,SAHS_NEW$depr_MS,graph = F)

##
## Original table
## SAHS_NEW$depr_MS
## SAHS_NEW$SAHSMS non oui Total
## 0 51 15 66
## 1 29 6 35
## Total 80 21 101
##
## Row percent
## SAHS_NEW$depr_MS
## SAHS_NEW$SAHSMS non oui Total
## 0 51 15 66
## (77.3) (22.7) (100)
## 1 29 6 35
## (82.9) (17.1) (100)
##
## Column percent
## SAHS_NEW$depr_MS
## SAHS_NEW$SAHSMS non % oui %
## 0 51 (63.7) 15 (71.4)
## 1 29 (36.2) 6 (28.6)
## Total 80 (100) 21 (100)

chisq.test(SAHS_NEW$SAHSMS,SAHS_NEW$depr_MS, correct = TRUE)

##
## Pearson's Chi-squared test with Yates' continuity correction
##
## data: SAHS_NEW$SAHSMS and SAHS_NEW$depr_MS
## X-squared = 0.16038, df = 1, p-value = 0.6888

SAHS_NEW$anxiete=as.factor(SAHS_NEW$anxiete)
tabpct(SAHS_NEW$SAHSMS,SAHS_NEW$anxiete,graph = F)

##
## Original table
## SAHS_NEW$anxiete
## SAHS_NEW$SAHSMS non oui Total
## 0 64 0 64
## 1 35 1 36
## Total 99 1 100
##
## Row percent
## SAHS_NEW$anxiete
## SAHS_NEW$SAHSMS non oui Total
## 0 64 0 64
## (100) (0) (100)
## 1 35 1 36
## (97.2) (2.8) (100)
##
## Column percent
## SAHS_NEW$anxiete
## SAHS_NEW$SAHSMS non % oui %
## 0 64 (64.6) 0 (0)
## 1 35 (35.4) 1 (100)
## Total 99 (100) 1 (100)

chisq.test(SAHS_NEW$SAHSMS,SAHS_NEW$anxiete, correct = TRUE)

## Warning in chisq.test(SAHS_NEW$SAHSMS, SAHS_NEW$anxiete, correct = TRUE): Chi-
## squared approximation may be incorrect

##
## Pearson's Chi-squared test with Yates' continuity correction
##
## data: SAHS_NEW$SAHSMS and SAHS_NEW$anxiete
## X-squared = 0.085929, df = 1, p-value = 0.7694

fisher.test(SAHS_NEW$SAHSMS,SAHS_NEW$anxiete)

##
## Fisher's Exact Test for Count Data
##
## data: SAHS_NEW$SAHSMS and SAHS_NEW$anxiete
## p-value = 0.36
## alternative hypothesis: true odds ratio is not equal to 1
## 95 percent confidence interval:
## 0.04558385 Inf
## sample estimates:
## odds ratio
## Inf

tabpct(SAHS_NEW$SAHSMS,SAHS_NEW$AVC, graph = F)

##
## Original table
## SAHS_NEW$AVC
## SAHS_NEW$SAHSMS 1 2 Total
## 0 23 50 73
## 1 15 22 37
## Total 38 72 110
##
## Row percent
## SAHS_NEW$AVC
## SAHS_NEW$SAHSMS 1 2 Total
## 0 23 50 73
## (31.5) (68.5) (100)
## 1 15 22 37
## (40.5) (59.5) (100)
##
## Column percent
## SAHS_NEW$AVC
## SAHS_NEW$SAHSMS 1 % 2 %
## 0 23 (60.5) 50 (69.4)
## 1 15 (39.5) 22 (30.6)
## Total 38 (100) 72 (100)

chisq.test(SAHS_NEW$SAHSMS,SAHS_NEW$AVC, correct = TRUE)

##
## Pearson's Chi-squared test with Yates' continuity correction
##
## data: SAHS_NEW$SAHSMS and SAHS_NEW$AVC
## X-squared = 0.53171, df = 1, p-value = 0.4659

SAHS_NEW$EPILEPSI=as.factor(SAHS_NEW$EPILEPSI)
tabpct(SAHS_NEW$SAHSMS,SAHS_NEW$EPILEPSI,graph = F)

##
## Original table
## SAHS_NEW$EPILEPSI
## SAHS_NEW$SAHSMS 1 2 Total
## 0 3 70 73
## 1 2 36 38
## Total 5 106 111
##
## Row percent
## SAHS_NEW$EPILEPSI
## SAHS_NEW$SAHSMS 1 2 Total
## 0 3 70 73
## (4.1) (95.9) (100)
## 1 2 36 38
## (5.3) (94.7) (100)
##
## Column percent
## SAHS_NEW$EPILEPSI
## SAHS_NEW$SAHSMS 1 % 2 %
## 0 3 (60) 70 (66)
## 1 2 (40) 36 (34)
## Total 5 (100) 106 (100)

chisq.test(SAHS_NEW$SAHSMS,SAHS_NEW$EPILEPSI, correct = TRUE)

## Warning in chisq.test(SAHS_NEW$SAHSMS, SAHS_NEW$EPILEPSI, correct = TRUE): Chi-
## squared approximation may be incorrect

##
## Pearson's Chi-squared test with Yates' continuity correction
##
## data: SAHS_NEW$SAHSMS and SAHS_NEW$EPILEPSI
## X-squared = 7.8066e-30, df = 1, p-value = 1

fisher.test(SAHS_NEW$SAHSMS,SAHS_NEW$EPILEPSI)

##
## Fisher's Exact Test for Count Data
##
## data: SAHS_NEW$SAHSMS and SAHS_NEW$EPILEPSI
## p-value = 1
## alternative hypothesis: true odds ratio is not equal to 1
## 95 percent confidence interval:
## 0.08457891 9.64542446
## sample estimates:
## odds ratio
## 0.7733186

SAHS_NEW$NEUROPSY="0"
SAHS_NEW$NEUROPSY[SAHS_NEW$depr_MS=="oui"|SAHS_NEW$anxiete=="oui"|SAHS_NEW$EPILEPSI=="1"|SAHS_NEW$AVC=="1"]="1"
SAHS_NEW$NEUROPSY=as.factor(SAHS_NEW$NEUROPSY)
tab1(SAHS_NEW$NEUROPSY,graph = F)

## SAHS_NEW$NEUROPSY :
## Frequency Percent Cum. percent
## 0 62 55.9 55.9
## 1 49 44.1 100.0
## Total 111 100.0 100.0

tabpct(SAHS_NEW$SAHSMS,SAHS_NEW$NEUROPSY,graph = F)

##
## Original table
## SAHS_NEW$NEUROPSY
## SAHS_NEW$SAHSMS 0 1 Total
## 0 42 31 73
## 1 20 18 38
## Total 62 49 111
##
## Row percent
## SAHS_NEW$NEUROPSY
## SAHS_NEW$SAHSMS 0 1 Total
## 0 42 31 73
## (57.5) (42.5) (100)
## 1 20 18 38
## (52.6) (47.4) (100)
##
## Column percent
## SAHS_NEW$NEUROPSY
## SAHS_NEW$SAHSMS 0 % 1 %
## 0 42 (67.7) 31 (63.3)
## 1 20 (32.3) 18 (36.7)
## Total 62 (100) 49 (100)

chisq.test(SAHS_NEW$SAHSMS,SAHS_NEW$NEUROPSY, correct = TRUE)

##
## Pearson's Chi-squared test with Yates' continuity correction
##
## data: SAHS_NEW$SAHSMS and SAHS_NEW$NEUROPSY
## X-squared = 0.085353, df = 1, p-value = 0.7702

fisher.test(SAHS_NEW$SAHSMS,SAHS_NEW$NEUROPSY)

##
## Fisher's Exact Test for Count Data
##
## data: SAHS_NEW$SAHSMS and SAHS_NEW$NEUROPSY
## p-value = 0.6889
## alternative hypothesis: true odds ratio is not equal to 1
## 95 percent confidence interval:
## 0.5133352 2.8822368
## sample estimates:
## odds ratio
## 1.217159

VIH

SAHS_NEW$VIH=as.factor(SAHS_NEW$VIH)
tabpct(SAHS_NEW$SAHSMS,SAHS_NEW$VIH,graph = F)

##
## Original table
## SAHS_NEW$VIH
## SAHS_NEW$SAHSMS 1 2 Total
## 0 7 66 73
## 1 1 37 38
## Total 8 103 111
##
## Row percent
## SAHS_NEW$VIH
## SAHS_NEW$SAHSMS 1 2 Total
## 0 7 66 73
## (9.6) (90.4) (100)
## 1 1 37 38
## (2.6) (97.4) (100)
##
## Column percent
## SAHS_NEW$VIH
## SAHS_NEW$SAHSMS 1 % 2 %
## 0 7 (87.5) 66 (64.1)
## 1 1 (12.5) 37 (35.9)
## Total 8 (100) 103 (100)

chisq.test(SAHS_NEW$SAHSMS,SAHS_NEW$VIH, correct = TRUE)

## Warning in chisq.test(SAHS_NEW$SAHSMS, SAHS_NEW$VIH, correct = TRUE): Chi-
## squared approximation may be incorrect

##
## Pearson's Chi-squared test with Yates' continuity correction
##
## data: SAHS_NEW$SAHSMS and SAHS_NEW$VIH
## X-squared = 0.91811, df = 1, p-value = 0.338

fisher.test(SAHS_NEW$SAHSMS,SAHS_NEW$VIH)

##
## Fisher's Exact Test for Count Data
##
## data: SAHS_NEW$SAHSMS and SAHS_NEW$VIH
## p-value = 0.26
## alternative hypothesis: true odds ratio is not equal to 1
## 95 percent confidence interval:
## 0.4695501 181.4155353
## sample estimates:
## odds ratio
## 3.887134

SAS CENTRAL

tab1(SAHS_HCY$SACS_Poka, graph = F)

## SAHS_HCY$SACS_Poka :
## Frequency Percent Cum. percent
## 0 105 94.6 94.6
## 1 6 5.4 100.0
## Total 111 100.0 100.0

tab1(SAHS_HCY[SAHS_HCY$SASSIGN=="1",]$SACS_Poka,graph = F)

## SAHS_HCY[SAHS_HCY$SASSIGN == "1", ]$SACS_Poka :
## Frequency Percent Cum. percent
## 0 55 90.2 90.2
## 1 6 9.8 100.0
## Total 61 100.0 100.0

tab1(SAHS_NEW[SAHS_NEW$IAH_MS=="1",]$SACS_Poka, graph = F)

## SAHS_NEW[SAHS_NEW$IAH_MS == "1", ]$SACS_Poka :
## Frequency Percent Cum. percent
## 0 39 88.6 88.6
## 1 5 11.4 100.0
## Total 44 100.0 100.0

tab1(SAHS_NEW[SAHS_NEW$SAHSMS=="1",]$SACS_Poka, graph = F)

## SAHS_NEW[SAHS_NEW$SAHSMS == "1", ]$SACS_Poka :
## Frequency Percent Cum. percent
## 0 33 86.8 86.8
## 1 5 13.2 100.0
## Total 38 100.0 100.0
